# Secretory microRNA-29 expression in gingival crevicular fluid during orthodontic tooth movement

**DOI:** 10.1371/journal.pone.0194238

**Published:** 2018-03-08

**Authors:** Phimon Atsawasuwan, Paul Lazari, Yinghua Chen, Xiaofeng Zhou, Grace Viana, Carla A. Evans

**Affiliations:** 1 Department of Orthodontics, University of Illinois at Chicago, Chicago, Illinois, United States of America; 2 Department of Periodontics, University of Illinois at Chicago, Chicago, Illinois, United States of America; 3 Department of Orthodontics, Boston University, Boston, Massachusetts, United States of America; Virginia Commonwealth University, UNITED STATES

## Abstract

Secretory microRNAs (miRNAs) have been used increasingly as biomarkers for cancers, autoimmune diseases and inflammatory diseases. They are reported as being freely circulated or encapsulated in microvesicles such as exosomes. This study was performed to elucidate the presence of miRNAs with exosomes in human gingival crevicular fluid (GCF), and the expression profile of *miRNA-29* during orthodontic tooth movement. Four healthy volunteer and fifteen orthodontic patients were enrolled in the study. Secretory miRNA in GCF was collected and analyzed using a bioanalyzer, realtime PCR and Western blot analysis. The expression profile of secretory *miR-29* family in GCF was analyzed during the course of canine retraction for 6 weeks. The results demonstrated the presence of miRNAs in the GCF. After series of ultracentrifugation and RT-PCR array, exosome-depleted fractions and pellets were isolated and we found that secretory miRNAs were detected in both the exosome-associated fraction and the exosome-depleted supernatant fraction; however, the concentration of miRNAs was higher in the exosome-associated fraction than in the exosome-depleted fraction suggesting a close association between the secretory miRNAs and exosomes in GCF. We also demonstrated the increased expression profiles of *miR-29* family during six weeks of orthodontic tooth movement in humans. Secretory miRNAs are present in GCF and secretory *miRNA-29* family expression profiles increase during the tooth movement in humans. Secretory *miRNA-29* in GCF could serve as potential biomarkers for periodontal remodeling.

## Introduction

MicroRNAs (miRNAs) are small non-coding RNAs, which are important in development, organogenesis, and homeostasis[1]. Several reports showed that miRNAs could be isolated from fresh or fixed tissues[2,3] and body fluids[4,5]. Secretory miRNAs have been isolated and shown to exist with remarkable stability in various types of body fluids[6]. The stability of secretory miRNA results from the formation of complexes between secretory miRNA and specific proteins[7]. Some secretory miRNAs were demonstrated to be packaged inside exosomes[8], which are small membranous vesicles about 30–100 nm in size and derived from the endosome[9]. The exosomes contain proteins and nucleic acids and can be secreted by many cell types[10]. The existence of secretory miRNAs in human serum and saliva has been reported to be concentrated in exosomes[4]. MiRNAs from unfractionated whole serum, urine, saliva, cerebrospinal fluid and exosomes could be promising biological tools as diagnostic biomarkers[8,11]. Several miRNAs play crucial roles in bone remodeling by controlling osteoblast/clast differentiation and function[12]. miRNAs-29a/b/c were involved in regulation of osteoblasts/clasts differentiation and could influence expression of certain extracellular matrix molecules i.e. collagens and their modifying enzymes[13–15]. Kagiya and Nakamura[16] found an increase in miRNA-29b during osteoclast differentiation in TNF-α/RANKL-treated cells, suggesting this miRNA plays a role in TNFα -regulated osteoclast differentiation. Franceschetti et al[17] found an increase in expression of all three members of miRNA-29 family during osteoclastogenesis, and repression of osteoclastogenesis process by inhibition of miRNA-29. The finding implicated that miRNA-29 promoted osteoclastogenesis.

Gingival crevicular fluid (GCF) is a serum transudate found in the gingival sulcus[18]. Irritation and inflammation of the gingival tissue increase the flow and alter the constituents of crevicular fluid. Serum is the primary source of the aqueous component of the GCF. The usual volume range of the GCF in the undisturbed sulcus is between 0.5–1μL[19]. A number of studies have measured cytokine and protein levels in GCF and analyzed this fluid to find biomarkers for several oral diseases such as gingivitis, periodontitis, root resorption and systemic diseases[20–22]. During orthodontic tooth movement (OTM), osteoclast plays a crucial role and its activities increases leading to alveolar bone resorption and tooth movement[23]. Changes in different substances found in GCF were reported in presence of orthodontic forces and various classes of molecules related to osteoclast activities such as Receptor activator of nuclear factor kappa-Β ligand (RANKL) and osteoprotegerin (OPG) contained in GCF have been reported as potential biomarkers for OTM[24,25]. This study we focused on miR-29 family due to their expression patterns in human periodontal ligament under loading[26] and their direct association with osteoclast function[16,17].This study was performed to elucidate the presence of miRNAs with exosomes in human gingival crevicular fluid (GCF), and the expression profile of *miRNA-29* during orthodontic tooth movement.

## Materials and methods

### Human subjects and GCF colllection

**Fig 1:**
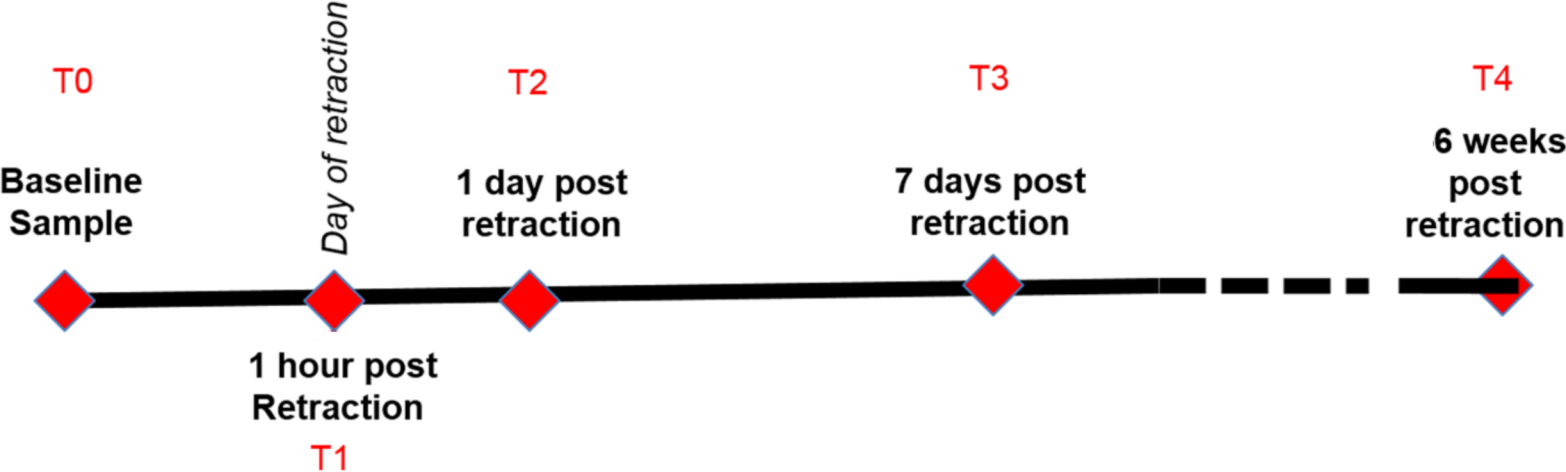
Diagram of human crevicular fluid collecting time. T0: prior to bonding the fixed orthodontic appliances, T1: on the day of canine retraction, 60 minutes after engaging the elastomeric powerchains onto the canine bracket. T2: 24 hours after initiation of canine retraction, T3: 7 days after initiation of canine retraction, T4: 6 weeks after initiation of canine retraction.

The study was approved by the Institutional Review Boards of the University of Illinois at Chicago (IRB protocol #2013–0183). All subjects and guardians signed a written informed consent and assent. To evaluate the presence of microRNA in gingival crevicular fluid (GCF), the GCF samples was collected from 4 healthy adult male volunteers aged 26–28 years old with excellent periodontal health. To study the profiles of secretory miR-29 family expression during maxillary canine retraction to close extraction spaces, seventy orthodontic patients aged 10–17 years old were screened and fifteen orthodontic patients was recruited for the study. Inclusion criteria were the following: (1) patients with excellent oral hygiene throughout the study period, (2) patients require at least maxillary first premolar extraction so canine retraction will be performed as a part of the treatment, (3) available to come back at the clinic at the times of sample collection. The exclusion criteria were (1) poor oral hygiene and/or bleeding on probing, (2) use medications, steroids or other anti-inflammatory medications, (3) systemic health problems or smoking. The collecting times were the following: T0: prior to bonding the fixed orthodontic appliances, T1: on the day of canine retraction, 60 minutes after engaging the elastomeric powerchains (American Orthodontics, Sheboygan,WI) onto the canine bracket. The powerchain for all the subjects was placed with an initial force of approximately 250g as measured by the Dontrix Force Gauge (Orthopli Corp, Philadelphia, Pa), T2: 24 hours after initiation of canine retraction, T3: 7 days after initiation of canine retraction, T4: 6 weeks after initiation of canine retraction. The timepoints of sample collection were shown in Fig 1.To collect the GCF samples, gingival sulcus of subjects was carefully isolated with cotton rolls to prevent a contamination from saliva. The GCF was collected by placing periopapers (Oraflow, Smithtown, NY) gently in gingival sulcus mesial to the tooth for 1 min. The collection process was repeated 4 times in the same location to collect adequate amount of GCF. The collected periopapers were stored in 200 μL cold sterile, RNase-DNase free phosphate buffered saline (PBS) and directly processed for the analysis.

### Exosome isolation

The samples from the 4 healthy individuals were centrifuged at 1,500g for 10 minutes to remove the periopapers and debris. The collected supernatant was centrifuged again at 17,000g for 15 minutes. An aliquot of the supernatant was subjected in an ultracentrifuged at 160,000g for 1 hour to isolate exosomes[4]. All centrifugations were performed at 4°C. The pellet containing exosomes and exosome-depleted supernatant were then processed for further experiments.

### RNA isolation and PCR array

Total RNA was extracted from all orthodontic patients’s total GCF samples and the 4 healthy subjects’ GCF samples, which were composed of non-ultracentrifuged supernatant, and exosome pellet and the exosome-depleted supernatant fractions after ultracentrifugation. miRNaeasy kit (Qiagen, Valencia, CA) was used for the total RNA isolation following the manufacturer’s instructions. The quality and quantity of the extracted RNA was assessed using an Agilent 2200 Tapestation bioanalyzer (Agilent Technologies, Santa Clara, CA) and NanoDrop 1000 spectrophotometer (Thermoscientific, Waltham, MA). For the Tapestation, 4μl R6K sample buffer and 1μl of RNA sample was mixed, heated at 72°C for 3 min then placed on ice for 2 min. Five microliters of DNA ladder and the samples were loaded into R6K screentape (Agilent Technologies,). The screentape was placed in the 2200 Tapestation and processed using the 2200 Tapestation Controller Software A.01.04 (Agilent Technologies). Quantitative real-time PCR (qPCR) was performed according to the manufacturer’s instructions (Qiagen) to profile the miRNA distribution in the exosome pellet and exosome-depleted-supernatant fractions of GCF. In brief, 5μl total RNA was used to generate cDNA using the miScript Reverse Transcription kit (Qiagen). The cDNA from each fraction was subject to miScript PCR array MIHS-001Z (Qiagen) to investigate the species and presence of secretory miRNAs in GCF. The RT-realtime PCR for quantification of miRNA-29 family was performed using commercially available primers (Life Technologies).

### Electron microscopy

To verify the presence of exosomes in GCF, the exosome pellet was fixed with cold 2% v/v glutaraldehyde in 0.1M PBS, rinsed in PBS, dehydrated through a graded series of ethanol and embedded in Epon. Ultra-thin sections (65nm) were stained with uranyl acetate and Reynold's lead citrate. A JEOL 100CX II transmission electron microscope was used for imaging.

### Western blotting

Proteins were extracted from the ultracentrifuged pellets and subject to SDS-PAGE then transferred to a PVDF membrane. The blotting membrane was blocked with skimmed milk and incubated with anti-human CD63 and CD9 antibodies (Abcam, Cambridge, MA) followed by incubation with horseradish peroxide-coupled secondary antibody (Abcam, Cambridge, MA). The proteins were detected using enhanced chemiluminescence (Thermo Scientific, Rockford, IL).

### Statistical analysis

The distribution of data was investigated using the Shapiro-Wilk test. Due to the distribution of the data and nature of the methods, Kruskal-Wallis statistical analyses were performed using IBM SPSS Statistics for Windows, V22.0, (Armonk, NY: IBM Corp.). Statistical significance was set at p ≤ 0.05. Descriptive statistical analysis was reported as mean + standard deviation.

## Results

### Secretory miRNAs were present in human gingival crevicular fluids

**Fig 2:**
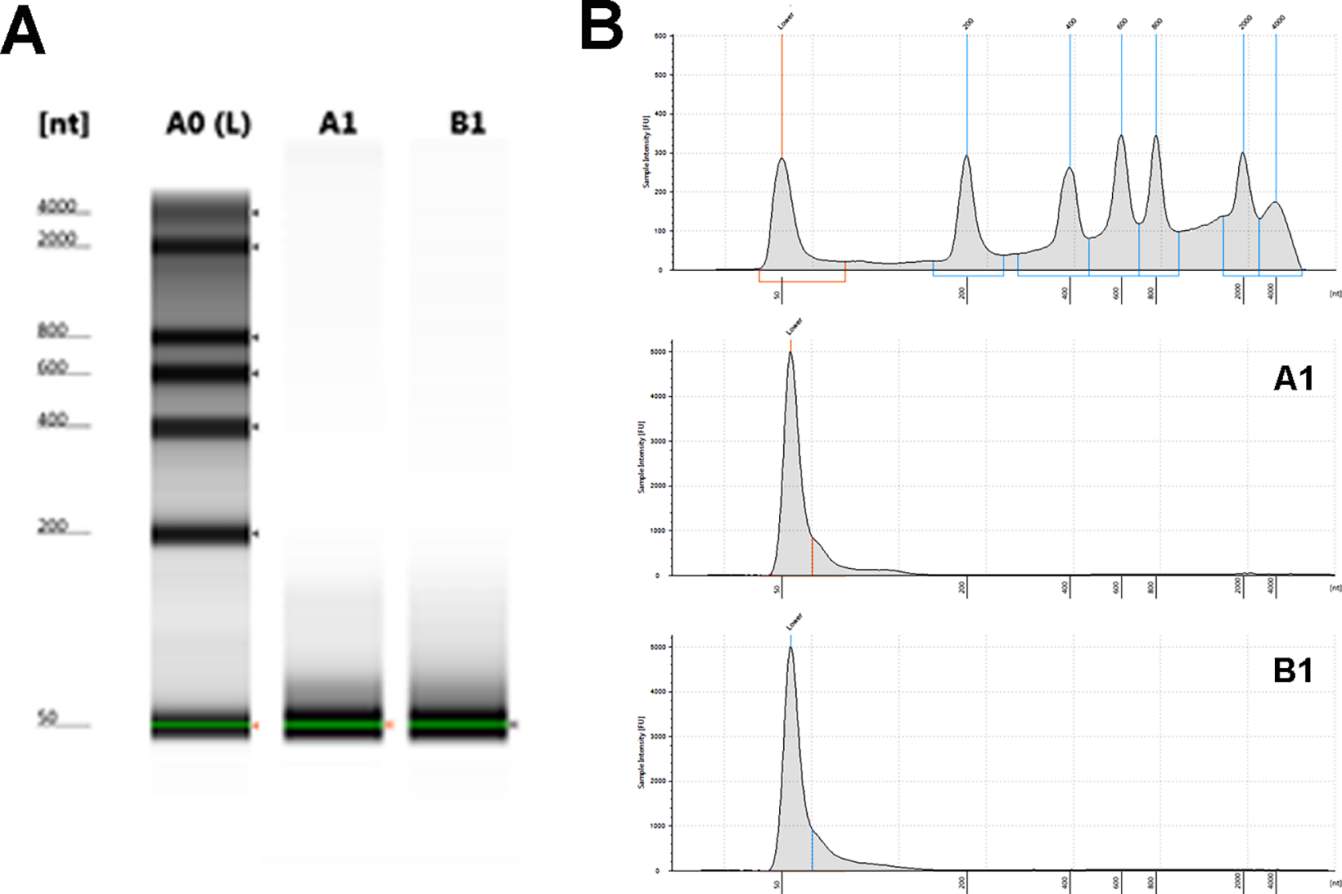
Presence of miRNA in healthy human crevicular fluid. “gel-like” images of R6K ScreenTape (A) after subjected to Tapestation bioanalyzer showed RNA ladder (L) on left lane and only small-sized RNA present in the sample lanes (middle and right lane). The positive bands approximately 50 nucleotides (nt) were present in representative GCF samples (A1 and B1). Electropherogram (B) corresponding to the gel-like images on the (A) figure. The *x*-axis on the electropherogram represents RNA size (nt), while the *y*-axis represents the measurement response of fluorescence units (FUs).

No subjects in this study had oral or periodontal pathology or were smokers. No pain or complication related to the noninvasive GCF collection using absorbent paper strips was reported. After the GCF collection from the 4 healthy subjects, the cell debris and paper strips were isolated from supernatant of the GCF by serial centrifugation at 1,500 and 17,000g. The supernatant was then subjected for total RNA isolation. After the total RNA isolation, examination of the isolated total RNA was immediately performed using a Tapestation 2200 and the results revealed that only small-sized of RNA were abundant in the collected samples (Fig 2A and 2B). No large-sized ribosomal RNA band was observed in any samples from bioanalyzer images (Fig 2B) indicating that there was no contaminated RNA from the cells or cell debris. The contaminated ribosomal RNA could not be removed simply by centrifugation. The existing small RNAs were endogenously present in the GCF.

### Exosomes were present in human gingival crevicular fluid

**Fig 3:**
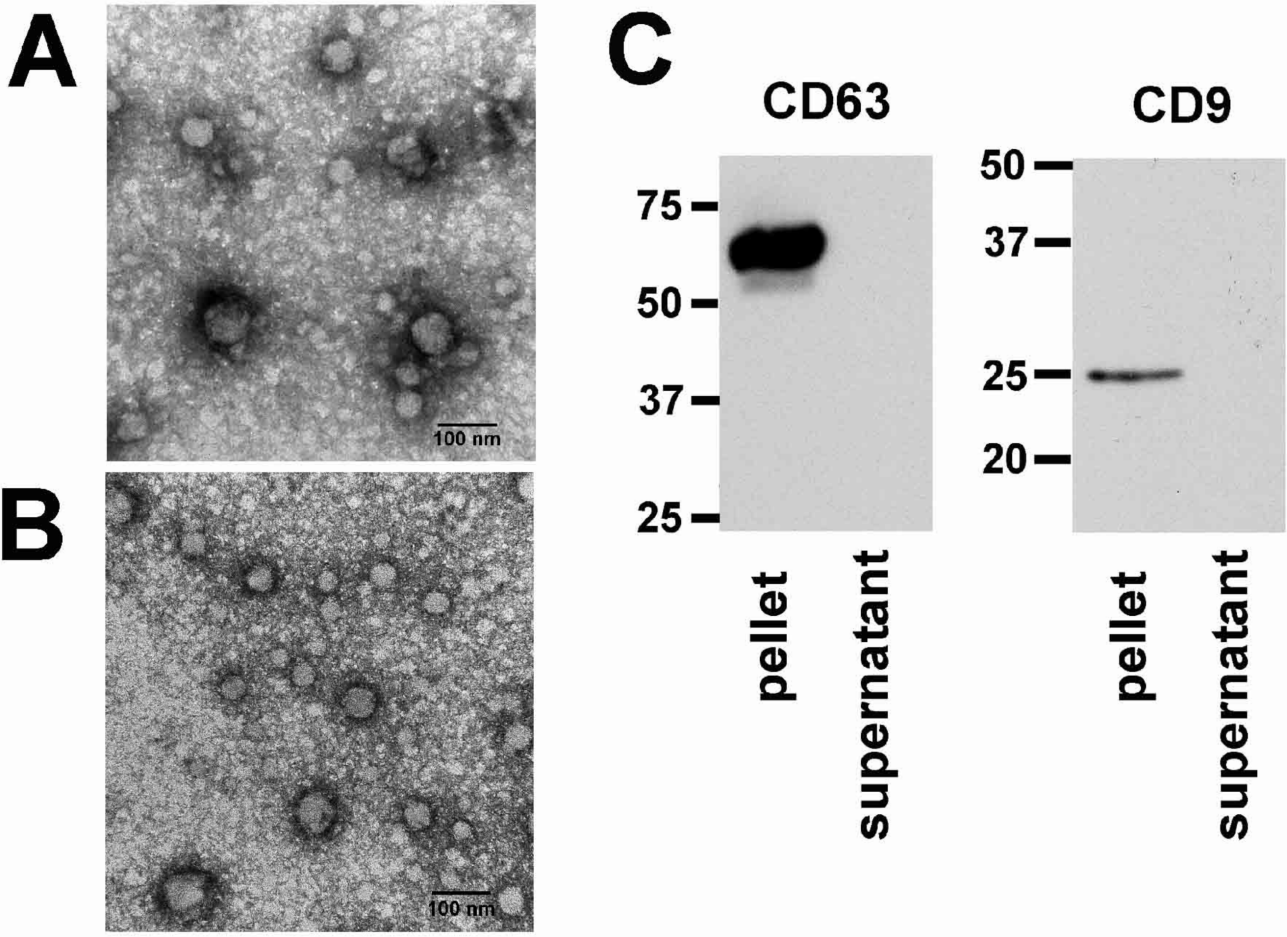
Electron microscope images and Western blot of isolated exosome from healthy human gingival crevicular fluid. Electron microscopy (A and B) of the ultracentrifugation pellet from GCF shows the characteristic spherical shape and size (50-100nm) of exosomes, Western blot (C) shows strong staining of the ultracentrifugation pellet with the exosomal membrane markers anti-CD63 and anti-CD9.

After ultracentrifugation, the fractions of collected GCF samples from the healthy subjects were subject to electron microscopic analysis. The pellet showed spherical structures varying in size between 50-110nm (Fig 3A and 3B) consistent with previously reported characteristics of exosomes[27]. The lysate of ultracentrifuged pellet and supernatant was subjected to Western blot analysis using antibodies against two commonly used exosomal markers, the tetraspanin molecules CD63 and CD9[28,29]. Immunostaining revealed that the exosome pellet fraction showed strong staining of CD63 and CD9 compared to negative staining of the exosome-depleted supernatant fraction (Fig 3C and S1 Fig).

### Majority of secretory miRNAs were associated with exosomes

**Fig 4:**
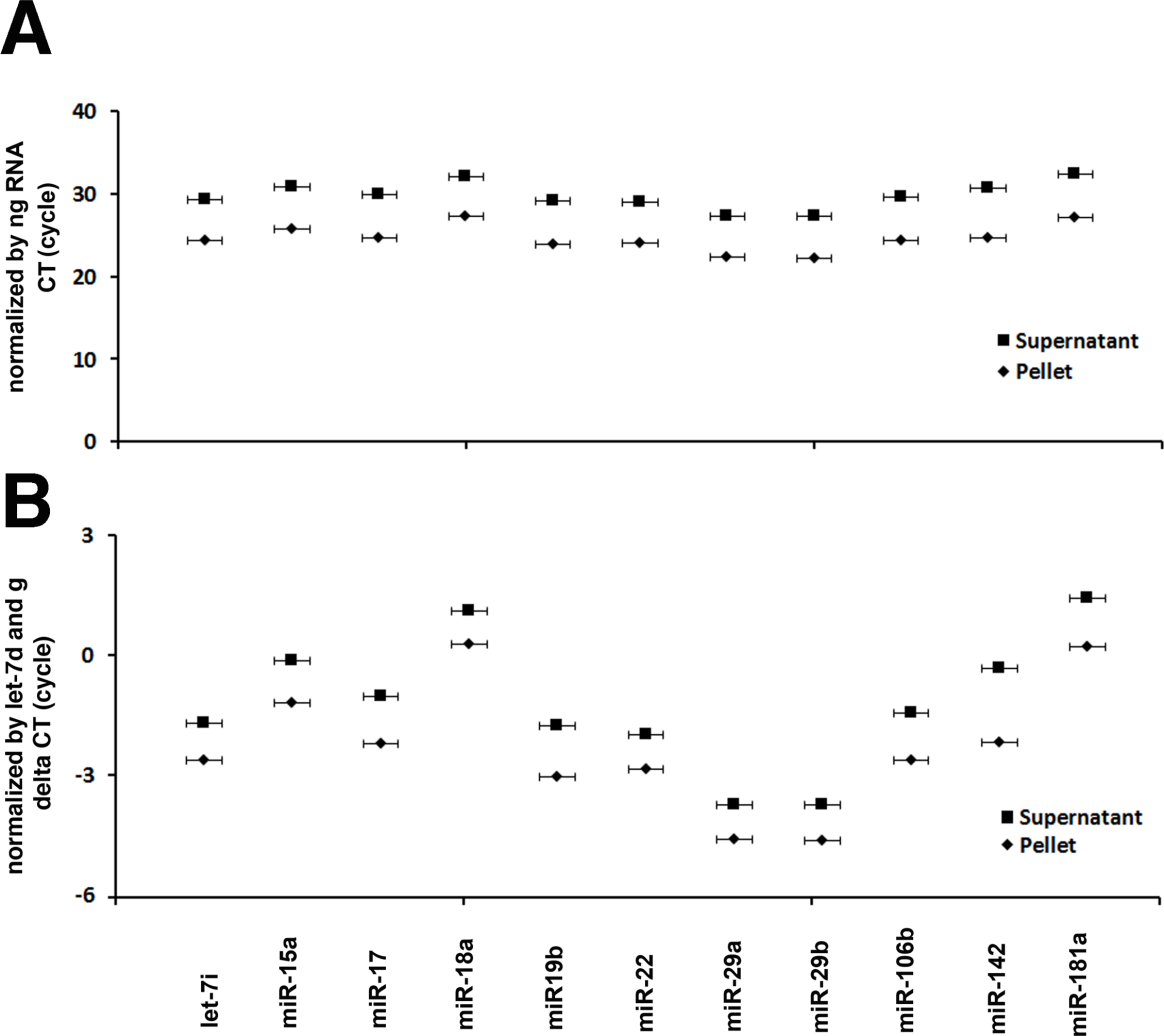
Distribution of miRNAs in exosome pellet and exosome-depleted supernatant fractions from crevicular crevicular fluid. Crevicular fluid miRNAs in all subjects are predominantly in exosomes; however, some miRNAs are present in exosome-depleted supernatant (A). The higher CT cycles are detected in exosome-depleted supernatant than in exosome pellet after normalized with amount of RNA (ng) (B) and the ΔCT is the difference between CT of supernatant-CT of exosome pellet after normalization with let-7d and g. Positive numbers show higher concentrations in the exosome pellet whereas negative numbers indicate higher concentrations in the exosome-depleted supernatant (C).

To quantify and determine whether miRNA in GCF is associated with exosomes or is freely circulated, we extracted the RNA from the exosome pellet fraction and from the exosome-depleted supernatant fraction. The whole exosomal pellet and entire volume of the supernatant were used for RNA isolation. Equal amounts of RNA in the pellet fraction and supernatant fraction were used for quantitative RT-PCR and subjected to miScript PCR array to examine species of secretory miRNAs in each fraction. The distribution of miRNAs in exosome pellet and exosome-depleted supernatant from each subject demonstrated that the majority of miRNAs detected in GCF was associated with the exosome pellet (S2 Fig). The expression levels of miRNAs in GCF were quantified and their amounts in exosome pellet and exosome-depleted supernatant were compared using quantitative RT-PCR. The RT-PCR revealed that the average number of CT of selected miRNAs in exosome pellet was about 5 cycles lower than the one in the supernatant (Fig 4A) and the same results were found when the CT of each miRNA was normalized using total amount of RNA in each fraction as internal control miRNAs (Fig 4B).

### Increased expression profiles of secretory miRNA-29 family were detected during canine retraction

**Fig 5:**
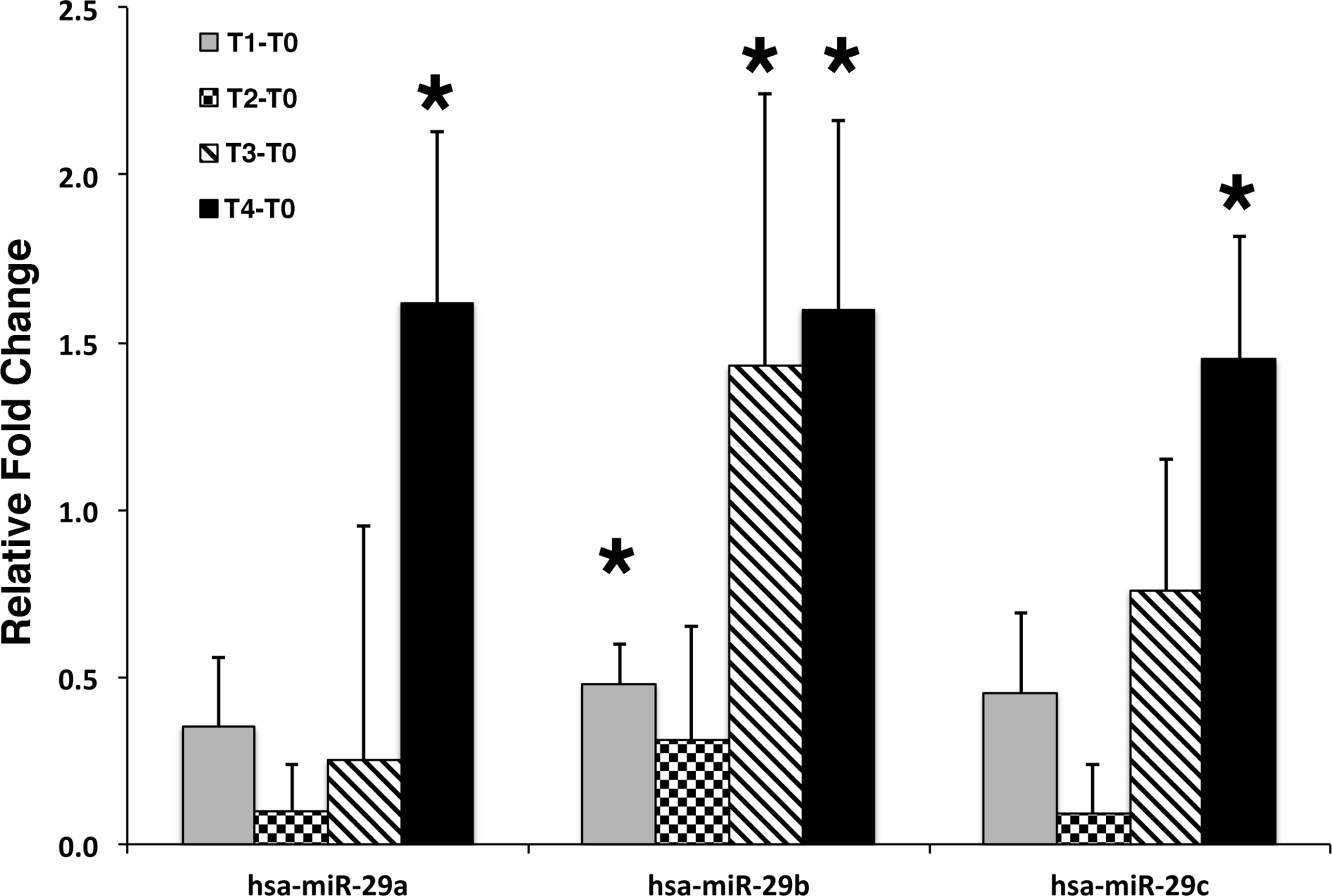
The expression profile of miRNA-29 family in GCF during tooth movement in human. The expression of miRNA-29a, -29b, and -29c were shown as gradually increase profile toward the last timepoint (6-wk). The significant differences are detected between T0 (pretreatment) and T4 (6-wk) in all studied miRNAs. Note that the significant differences of miRNA-29b expression are detected between T0 and T1 (1-hr), T0 and T3 (7-day) (P<0.05).

Due to the limited quantity of GCF sample collected from a single canine during the canine retraction, the total GCF sample not fractionated sample from each canine was tested in this part of study. Along the course of canine retraction, after normalization with internal miRNA controls (Let-7d, g and i), the change in miRNA-29 family expression patterns from pretreatment (T0) to 6 weeks postretraction (T4) showed statistical significance consistently (P<0.05). The expression patterns of miR-29 family expression demonstrated that the expression levels were spiked upward immediately after retraction, leveled off 1 hour postretraction (T1), and then gradually increased until the end of study (T4) (Fig 5). Significant changes were found between T0 and T4 in all of the miRNA-29 family however, the significant changes between T0 and 1 hour postretraction (T1), and T0 and 7-d postretraction (T3) were only observed in miRNA-29b (P<0.05) (Fig 5). Analyzing all the time points together, no statistical significant difference was found between miRNA-29a, miRNA-29b and miRNA-29c (P>0.05) indicating the similarity in profile changes of the miRNA-29 family. The fold change from ΔT0-T4 of miRNA-29a, b and c were 1.45+0.37 to 1.62+0.51 (Table 1).

**Table 1:** Relative fold change (ΔT) of each studied microRNA at different timepoints.

	T0-T1		T0-T2		T0-T3		T0-T4	
	Fold change	SE	Fold change	SE	Fold change	SE	Fold change	SE
29a	0.35	0.21	0.10	0.14	0.25	0.70	1.62*	0.51
29b	0.48*	0.12	0.31	0.34	1.43*	0.81	1.60*	0.56
29c	0.45	0.24	0.09	0.15	0.76	0.39	1.45*	0.37

* P<0.05; T0: baseline; T1: 1hr postretraction, T2: 24hr postretraction, T3: 7d postretraction, T4: 6wk postretraction.

## Discussion

This study demonstrates the presence of secretory miRNA in gingival crevicular fluid (GCF) and that the exosome fraction of the GCF is highly enriched in miRNAs. That the secretory miRNAs detected in GCF are associated primarily with exosomes is similar to the previous finding of miRNAs in serum and saliva[4]. Though the majority of miRNAs in GCF is associated with exosomes, the amount in whole GCF is not as large as serum or saliva and this limitation would limit the potential of exosome seperation. Because the origin of GCF is serum, the nature and existence of secretory miRNAs in GCF would be similar to the ones in serum[4]. Normalization of secretory miRNAs, a crucial step in determining changes in expression of miRNA between samples, is necessary to remove variation between samples and isolate the change due to treatment effect[30]. There are traditionally used housekeeping genes that are used in quantitative studies of miRNA, depending on the source medium of the miRNA. Since there was no previous comparative study that analyzed miRNA in GCF, a serum derivative, we selected a group of miRNAs (Let-7d, Let-7g, Let-7i) that had shown stable expression level in various serum samples and were reported to be suitable options to be used as internal control for serum miRNA normalization[31]. Recently a pilot study showed the presence of miRNA in crevicular fluid of periodontitis patients[32]. However, the present study was the first to report the change of secretory miRNA expression profile in gingival crevicular fluid during tooth movement in humans. We reported that different orientation of forces affected the expression pattern of miRNA-29 in human periodontal ligament cells[26]. MiRNA-29 family (a/b/c) were selected for this study based on their potential involvement in molecular and cellular pathways associated with OTM. In our laboratory, miR-29 sponge mouse demonstrated delayed tooth movement with reduced numbers of osteoclasts (data not shown). Recent studies have shown that miRNAs-29a/b/c were involved in osteoclast regulation and could influence expression of certain extracellular matrix molecules i.e. collagens and their modifying enzymes[13–17]. Previous reports showed an increase in miRNA-29b during osteoclast differentiation in TNF-**α**/ RANKL-treated cells[16] and promoted osteoclastogenesis by regulating osteoclast commitment and migration[17]. During OTM, several reports reported the activity of osteoclast using expression level ratio of Receptor activator of nuclear factor kappa-B ligand (RANKL) and osteoprotegerin (OPG) in GCF. The activity of osteoclasts was not significantly increased until day 7 of force application followed by significantly increased after 42 days of force application[33,34]. Interestingly the expression profiles of miRNA-29 family in this study were similar to the reported activity of osteoclasts as their expression significantly increased after day 35 of canine retraction implicating secretory miRNA-29 family expression in GCF were associated with osteoclast activity during the tooth movement.

We demonstrated that secretory miRNAs were present in GCF and seems to be associated with exosomes. In addition, expression of secretory specific miRNAs such as miRNA-29 family seems to be correlated with osteoclast function suggesting the potential of secretory miRNA-29 family during the canine retraction.

## Supporting information

S1 Fig Full gel Western blot of CD63 and CD 9 in exosomal precipitate and exosome depleted supernatant fraction. CD9 and CD63 were positive only in exosomal precipitate fraction and no exosome in the supernatant fraction.
(TIF)

S2 Fig Distribution of each miRNA in each fraction of samples after subjected to the miScript PCR array MIHS-001Z. Majority of miRNAs were identified in the exosome pellet fraction (red color) while only minority of miRNAs were identified in the supernatant fraction (green color) and some of miRNAs could be detected in both fractions (black color).
(TIF)
