# Supplementary figures and images for: Secretory microRNA-29 expression in gingival crevicular fluid during orthodontic tooth movement
Source: PLoS One. 2018 Mar 8;13(3):e0194238. doi: 10.1371/journal.pone.0194238 (PMC5843286; doi:10.1371/journal.pone.0194238)

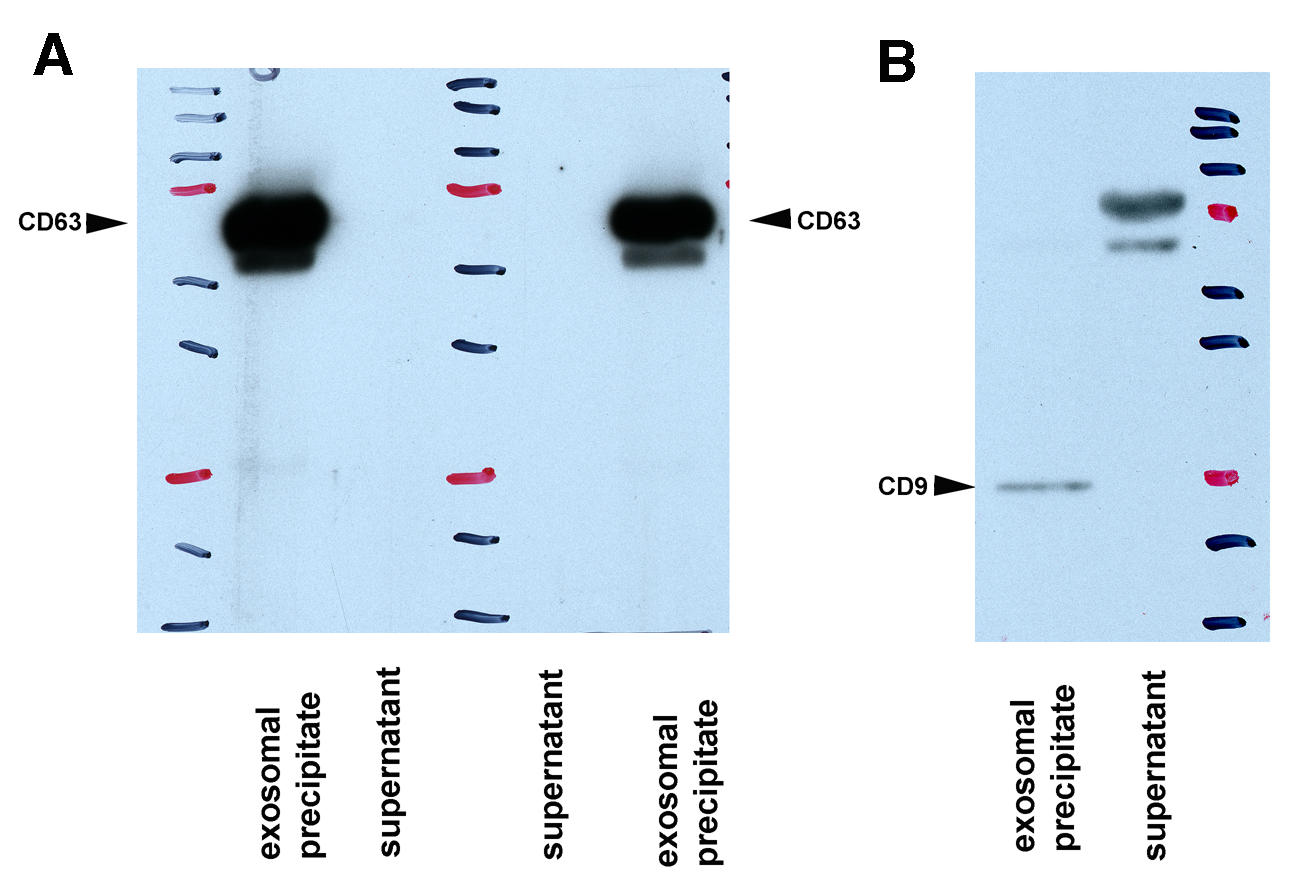

Supplement: S1 Fig — CD9 and CD63 were positive only in exosomal precipitate fraction and no exosome in the supernatant fraction. (TIF) [file pone.0194238.s001.tif]

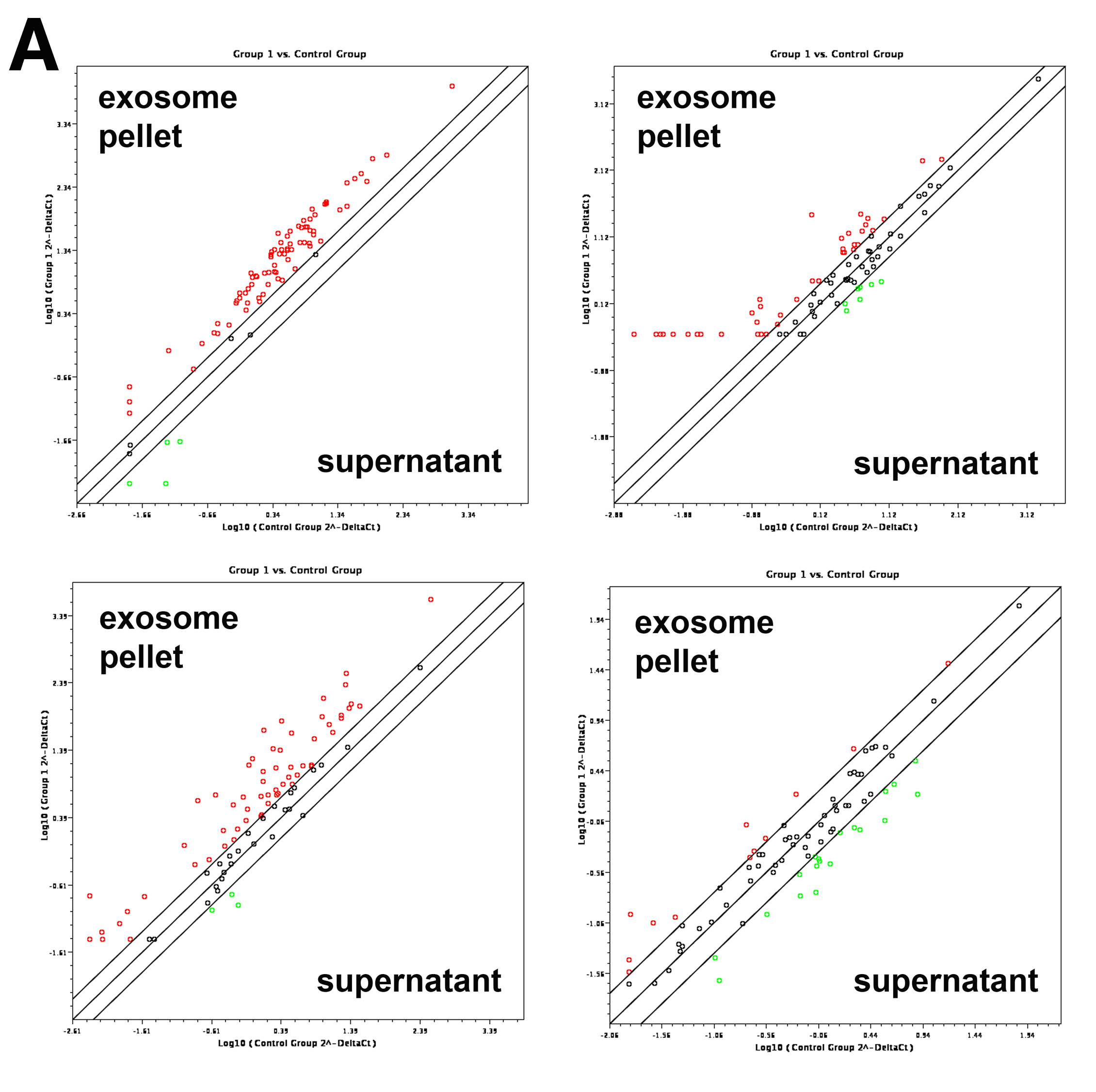

Supplement: S2 Fig — Majority of miRNAs were identified in the exosome pellet fraction (red color) while only minority of miRNAs were identified in the supernatant fraction (green color) and some of miRNAs could be detected in both fractions (black color). (TIF) [file pone.0194238.s002.tif]
